# Epileptic networks neurosurgery: connectomes and hubs

**DOI:** 10.1093/lifemedi/lnac033

**Published:** 2022-09-02

**Authors:** Kailiang Wang, Penghu Wei, Yongzhi Shan, Guoguang Zhao

**Affiliations:** Department of Neurosurgery, Xuanwu Hospital Capital Medical University, Beijing 100053, China; China International Neuroscience Institute (China-INI), Beijing 100053, China; China National Medical Center for Neurological Diseases, Beijing 100053, China; Clinical Research Center for Epilepsy, Capital Medical University, Beijing 100053, China; Department of Neurosurgery, Xuanwu Hospital Capital Medical University, Beijing 100053, China; China International Neuroscience Institute (China-INI), Beijing 100053, China; China National Medical Center for Neurological Diseases, Beijing 100053, China; Clinical Research Center for Epilepsy, Capital Medical University, Beijing 100053, China; Department of Neurosurgery, Xuanwu Hospital Capital Medical University, Beijing 100053, China; China International Neuroscience Institute (China-INI), Beijing 100053, China; China National Medical Center for Neurological Diseases, Beijing 100053, China; Clinical Research Center for Epilepsy, Capital Medical University, Beijing 100053, China; Department of Neurosurgery, Xuanwu Hospital Capital Medical University, Beijing 100053, China; China International Neuroscience Institute (China-INI), Beijing 100053, China; China National Medical Center for Neurological Diseases, Beijing 100053, China; Clinical Research Center for Epilepsy, Capital Medical University, Beijing 100053, China

We propose that current treatment of epilepsy should be centered on brain networks and epilepsy networks, and that epilepsy surgery should be developed under the auspices of the newly emerging branch epileptic networks neurosurgery (ENN), based on the rapidly developing theory of human connectomes and brain network hubs [1, 2]. There are three reasons that support the necessity for this new direction. First, recent studies have characterized epilepsy as essentially a neural network disease that originates from one or multiple epileptic foci and propagates through specific epileptic circuits [2]. These circuits are usually referred to as the epilepsy network or epileptic connectomes, and the epileptic foci can be called epileptic hubs. Second, recent progress in analyzing and decoding the brain functioning mechanism has resulted in a powerful research tool: the brain network theory system, also called connectomes theory [1]. An increasing number of neuroimaging and neurophysiological data modalities have been used to interpret how the brain works, among which resting-state functional MRI is the most commonly used data modality to study brain functional connectivity. In terms of structural connectivity, tractography-based diffusion tensor imaging (DTI)/diffusion spectrum imaging (DSI) data, cortical thickness and volume covariance have been adapted to analyze brain structural wiring. In addition, high-temporal resolution neurophysiological data have been used to decode the instantaneous information flow in the brain. The third reason for the adoption of ENN is that traditional epileptic-focus resective operations cannot completely control epilepsy symptoms. As reported, the success rate of epileptic-focus resection operations decreases and the number of seizure-free patients is reduced 10-year postoperatively [3]. Furthermore, these resective operations may cause damage to normal brain function, such as memory loss, cognitive impairment or limb movement disorders.

Because of the above issues, new therapeutic concepts for advancing epilepsy treatment options [4] are now taking hold that are more concordant with network theory, including radiofrequency thermocoagulation (RFTC), laser interstitial thermotherapy (LITT), deep brain stimulation (DBS), responsive neurostimulation (RNS), and even some noninvasive neuromodulation methods such as transcranial current stimulation (TCS) and transcranial magnetic stimulation (TMS). These methods aim to control the seizure or other epileptic manifestations by modulating or disrupting the key nodes (epileptic hubs) and thus normalize the aberrant epilepsy-related brain networks. According to the brain network theory, the brain can be modeled as a complex pattern of spatial-temporal connections consisting of “nodes” and connected “edges,” i.e. the connectomes [1]. In this complex system, some nodes play vital roles in maintaining connectome stability and function and are called “hubs.” Mounting evidence indicates that when the hubs are disrupted, the connectomes become unbalanced and their connectivity is disturbed; in human brain, this will be manifested as various pathological symptoms, e.g. seizure.

Currently, the most widely used research method to understand complex networks is graph theory [1], which enables the computation of a series of data-driven network topological measures such as degree centrality, betweenness centrality, clustering coefficients, modularity, rich club, and others. These network parameters can be used to illustrate the brain functional state with different topological properties and organizational structures in both healthy and pathologic populations. In particular, compared with controls, hub nodes are often identified using significant network metrics in graph theory, e.g. the 5%–10% top-ranked nodes, based on degree centrality or betweenness centrality. The localization of the epileptogenic focus has always been the most critical step in epilepsy preoperation evaluation and directly affects surgical strategy decision-making. In line with connectomes theory, the localization of the epileptogenic focus is equivalent to finding the hubs in the epileptic network. With advancing imaging and neurophysiological techniques, especially for MRI-negative epilepsy, many focus detection algorithms have been adopted. Morphological calculations for MRI structural imaging are based on voxel or vertex data, including volume calculation, surface thickness, cortical deformation, curvature, sulcal depth, gray matter/white matter contrast, junction images, and extension images. DTI/DSI based methods include fiber tractography, relative anisotropy, and mean diffusivity; meanwhile, metabolic-based PET or SPECT data analysis is aimed at finding the epilepsy-associated hypometabolic zone, while functional MRI methods include amplitude of low-frequency fluctuation, regional homogeneity (Reho), and other techniques. Another aspect is the rapid advancement in neurophysiological technology. In addition to the classical scalp EEG and subdural ECog, the SEEG technique has been widely used to detect small or occult lesions with both high temporal and spatial resolution simultaneously, including local field potential-related high frequency oscillations (HFO), low-amplitude fast rhythm and suppression of low frequencies. In terms of connectomes theory, all of the above-mentioned algorithms can be used to define the hub nodes in the connectomes, which would then be listed as the suspicious epileptic focus, the storm center of the epileptic connectomes.

The most important aspect of the ENN system is the change in the concept of epilepsy treatment: from classic focal resection of the “epileptogenic focus” to the evaluation of the entire epileptic network, which is aimed at inhibiting propagation through the epilepsy network and replaces the epileptogenic foci hypothesis with epileptic connectomes. As for clinical practice, the change to the ENN system first underscores the importance of epileptic hub regions and expands the study of epilepsy treatment to a focus on epileptic neural circuits, namely, the epileptic connectomes. Second, it highlights the role of noninvasive and micro-invasive innovative techniques in network interventions, i.e. reconfiguration, modulation, or intervention of hub nodes or regions in epilepsy connectomes through DBS, RNS, TCS, or TMS [4]. Third, ENN will be combined with the artificial intelligence technology to simulate surgical resection, brain stimulation, or neuromodulation of the hub regions to verify the accuracy and precision of surgical decision-making, thus resulting in minimally invasive treatment of epilepsy.

**Figure 1.**
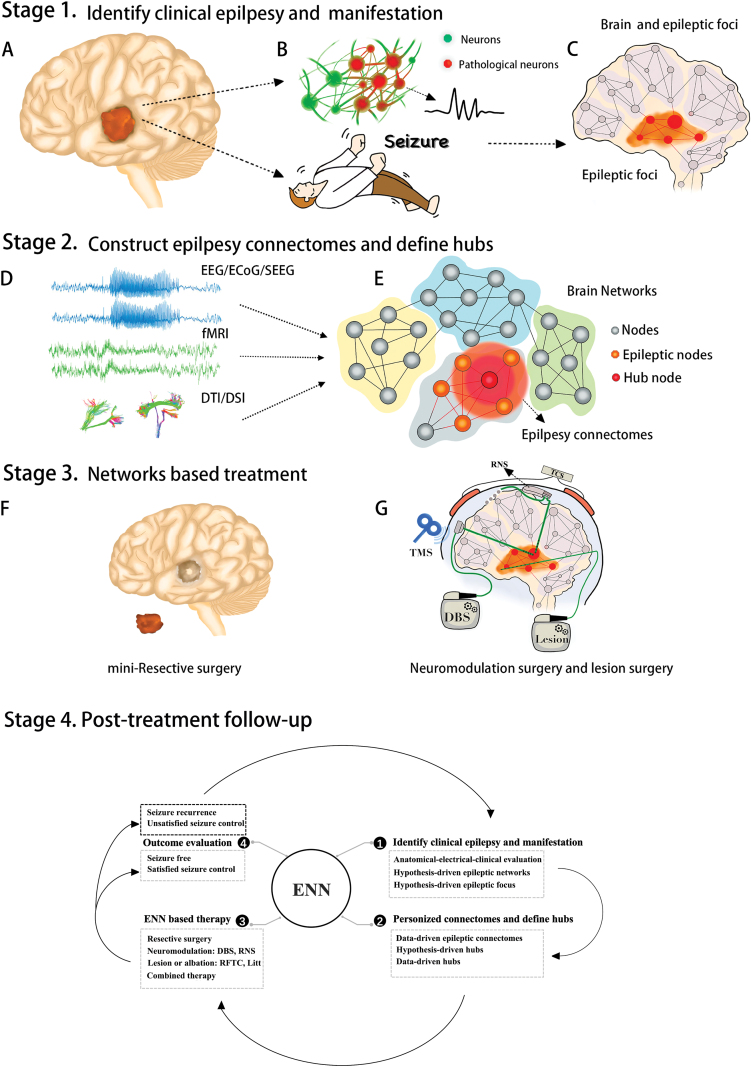
Illustration of ENN. We suggest the illustrated protocol, which divides the implementation of ENN into four stages. (1) Stage 1: Identify clinical epilepsy and manifestations. A multidisciplinary team is needed to perform the presurgical evaluation, identifying the suspicious epileptogenic foci whether the MRI is positive or negative [as shown in (A), MRI-positive] and determining possible pathologic types (such as focal cortical dysplasia, tumor, or hippocampal sclerosis) and related clinical symptoms, to achieve concordance of symptomatologic localization and epileptogenic focus localization [as shown in (B)]; and constructing the hypothetical brain circuits (C) of the origin and propagation of epilepsy. (2) Stage 2: Construct the epilepsy connectomes and define hubs. Multimodal imaging and neurophysiological data are necessary to establish the brain networks and epileptic connectomes, utilizing fMRI, structural DTI/DSI, SEEG, ECog, and EEG data, as shown in (D). Mapping of the epileptic manifestation to the hub nodes is then performed, as illustrated in (E). In the established the brain networks, it is important to identify the epilepsy connectomes and hub nodes. As for the epilepsy connectomes, we suggest use of independent component analysis or seeds-based methods (derived from stage 1 and neurophysiological data) to separate the epilepsy networks from whole brain networks. Then, for the identification of hub nodes, epileptic nodes, and common nodes, data-driven graph methods should be used to define the hub nodes, such as degree centrality, betweenness centrality, structural quantitative analysis algorithms, or other network measures, as shown in (E). Furthermore, hypothesis driven measures should also be considered, according to the anatomical–electrical–clinical interpretation. (3) Stage 3: ENN-based treatment. Once the epilepsy connectomes and hubs nodes are identified, ENN-based treatment can be performed mainly focused on the hub nodes; first, compared to conventional excisional surgery, the resected regions will be more limited to the hub nodes (F) and mini-resective surgery more precise and minimally invasive. Second, neuromodulation methods will play a more important role in ENN, such as the invasive DBS, RNS, and noninvasive TCS (direct or alternating) and TMS. Other options are the lesion approach, including SEEG-RFTC and LITT, as shown in (G). (4) Stage 4: Posttreatment follow-up. After the ENN-based treatment, follow-up of patients with seizure free or satisfied seizure control could quit the ENN-Loop; otherwise, for patients with unsatisfied seizure control or recurrence should re- the ENN-Loop again and be re-evaluated, also enter the above-mentioned stages again.

According to ENN, in clinical practice, treatment should be performed as follows: (i) During the preoperation evaluation, in addition to the traditional multidisciplinary team members with background in neurosurgery, neuropsychology, neuroimaging, and neurology, team members should also be employed with expertise in the fields of neural engineering, big data, and mathematical models to form a medical-engineering team specialized in the building, analyzing, interpreting, simulating, and modulating of brain networks. (ii) Presurgical evaluation of epileptic connectomes and mapping the epileptic manifestation to the hub nodes will play a critical role in decision-making. Therefore, how to construct an individual precise epileptic connectome and how to define the hub epileptic nodes in the current connectome should be closely linked to the prognosis of the epilepsy patient. According to our experience, multimodal imaging data (from at least two imaging methods including functional MRI, structural MRI, DTI/DSI, PET, or FDG-PET) and neurophysiological data (including data from at least one of SEEG, ECog, or EEG) are both essential for constructing the epileptic connectomes. As to the hub mapping methods, in addition to network data-driven measures derived from graph theory (degree centrality, betweenness centrality, clustering coefficient, modularity, etc.), digital analytical algorithms (e.g. HFO or Reho) for detecting the aberrant MRI regions or abnormal neurophysiological frequency, power or other nonlinear features, as well as anatomical hypothetical methods based on clinical semiology, should all be included to define the hub nodes [2]. To determine the specific epileptic involvement of regions, cortical cortex, and subcortical structures (thalamus, caudate, or other nuclei) are equally important. In these cases, SEEG would be the most powerful tool to probe the seizure connectomes with intraoperation intracranial monitoring or low-frequency stimulation. (iii) Surgical treatment strategy of epilepsy, in addition to conventional excisional surgery, should also be combined with a variety of noninvasive and minimally invasive treatments, including SEEG-RFTC, LITT, DBS, RNS, TCS, and rTMS. In addition, the strategy of upfront combinatorial therapy is recommended for some complex patients, when the treatment could be completed in stages; e.g. for seizure control, resective operation or lesion/ablation could be used first, then continued with the neuromodulation approach (DBS or RNS) 6 months or 1 year later. In this case, we have suggested a protocol that divides the implementation of ENN into four stages, as illustrated in Fig. 1. According to ENN-Loop, with the goal of seizure-free or satisfied seizure control, the treatment of epilepsy is not once and for all, but repeated and lasting.

As a newly emerging branch of neurosurgery, the establishment and development of ENN centered on epileptic connectomes and hubs is both necessary and promising. Any new concept will face challenges and require supporting evidence from long-time clinical practice. There are still many challenges which need to be resolved in ENN, including the standardization of methods for constructing epilepsy connectomes, the precise definition of the hub nodes in the network, the reliability of models for simulating intervention techniques in the network, and the reproducibility of the epileptogenic network based on individualized data. With advances in artificial intelligence, machine learning, and deep learning technologies, as well as development of digital virtual brains, new multimodal imaging, and neurophysiological techniques, we believe the technical difficulties for ENN will gradually be solved. On the other hand, at the microscopic level, the in-depth study of epilepsy gene networks, molecular protein networks, signaling pathway networks, and other precise microscopic network scales will also provide theoretical support for the future development of ENN [5].
